# ‘It struck at the heart of who I thought I was’: A meta‐synthesis of the qualitative literature examining the experiences of people with multiple sclerosis

**DOI:** 10.1111/hex.13093

**Published:** 2020-06-24

**Authors:** Jane Desborough, Crystal Brunoro, Anne Parkinson, Katrina Chisholm, Mark Elisha, Janet Drew, Vanessa Fanning, Christian Lueck, Anne Bruestle, Matthew Cook, Hanna Suominen, Antonio Tricoli, Adam Henschke, Christine Phillips

**Affiliations:** ^1^ Department of Health Services Research and Policy Research School of Population Health College of Health and Medicine Australian National University Canberra ACT Australia; ^2^ Australian National University Medical School College of Health and Medicine Australian National University Canberra ACT Australia; ^3^ John Curtin School of Medical Research College of Health and Medicine Australian National University Canberra ACT Australia; ^4^ School of Computer Science College of Engineering and Computer Science Australian National University Canberra ACT Australia; ^5^ Research School of Electrical Energy & Materials Engineering College of Engineering and Computer Science Australian National University Canberra ACT Australia; ^6^ National Security College College of Asia and the Pacific Australian National University Canberra ACT Australia

**Keywords:** MS, multiple sclerosis, patient experience, perceptions, review literature

## Abstract

**Background:**

People with multiple sclerosis (MS) have varied experiences and approaches to self‐management. This review aimed to explore the experiences of people with MS, and consider the implications of these experiences for clinical practice and research.

**Methods:**

A meta‐synthesis of the qualitative literature examining experiences of people with MS was conducted using systematic searches of ProQuest, PubMed, CINAHL and PsycINFO. We incorporated feedback from team members with MS as expert patient knowledge‐users to capture the complex subjectivities of persons with lived experience responding to research on lived experience of the same disease.

**Results:**

Of 1680 unique articles, 77 met the inclusion criteria. We identified five experiential themes: (a) the quest for knowledge, expertise and understanding, (b) uncertain trajectories (c) loss of valued roles and activities, and the threat of a changing identity, (d) managing fatigue and its impacts on life and relationships, and (f) adapting to life with MS. These themes were distributed across three domains related to disease (symptoms; diagnosis; progression and relapse) and two contexts (the health‐care sector; and work, social and family life).

**Conclusion:**

The majority of people in the studies included in this review expressed a determination to adapt to MS, indicating a strong motivation for people with MS and clinicians to collaborate in the quest for knowledge. Clinicians caring for people with MS need to consider the experiential and social outcomes of this disease such as fatigue and the preservation of valued social roles, and incorporate this into case management and clinical planning.

## 1 INTRODUCTION

Multiple sclerosis (MS) is one of the most common inflammatory neurological conditions and a major cause of non‐traumatic neurologic disability among younger adults.^1^ Worldwide, more than 2.2 million people, mostly female, are estimated to be affected.^1^ MS varies in its presentation, clinical course and the frequency and severity of symptoms experienced. Many people present initially with a relapsing‐remitting form of the disease, characterized by symptom‐free periods and recovery which follow attacks or relapses.^2^ For others, MS begins as a primary progressive form, or develops into secondary progressive MS, with gradual worsening of neurological symptoms and increasing disability over time.^2^

Although several risk factors have been identified, the cause of MS remains unknown and to date, there is no known cure.^2^, ^3^ Many disease‐modifying therapies are available that can reduce symptoms and relapse frequency, with the ultimate aim of preventing all disease activity.^4^ Most of these treatments modify immunity and are administered variously via oral, intramuscular, subcutaneous and intravenous routes. All treatments carry risk of side effects, including pervasive flu‐like symptoms as a direct consequence of treatment (type 1 interferons), heightened susceptibility to infections as a result of immune suppression, and drug hypersensitivity and injection site reactions,^5^ which can impact people's willingness to use them.^6^ Overall, the relationship between therapies and disease outcomes is uncertain for any particular person, as is the range of side‐effects a person may experience.

Perhaps because of the heterogeneity of disease experiences of MS, the literature has tended to atomize, rather than synthesize these experiences. Qualitative studies have focused on the experiences of people with MS at particular points in time (eg diagnosis, early stage and relapse),^7^, ^8^ in specific populations (eg women and mothers),^9^, ^10^, ^11^, ^12^ in relation to specific assessments or interventions (eg rehabilitation, physical activity, disease‐modifying therapies or alternative therapies),^13^, ^14^, ^15^, ^16^, ^17^ or of specific symptoms or consequences (eg fatigue or sexual dysfunction).^18^, ^19^, ^20^

The purpose of this review was to: (a) conduct a systematic search of the published qualitative literature on the experiences of people with MS; (b) synthesize the results to elucidate the common impacts of MS on people's lives; and (c) discuss these experiences in relation to clinical practice and research.

## 2 METHODS

We used the scoping review approach described by Arksey and O'Malley^23^ and enhanced by Levac et al^24^ and involved six stages: (a) identifying the research question, (b) identifying relevant studies, (c) selecting studies, (d) charting the data, (e) collating, summarizing and reporting the results, and (f) consulting with relevant stakeholders. Our collation and summation of the results involved arriving at a consensus of the overarching themes derived from the included studies and a meta‐synthesis of these.

The multidisciplinary research team involved in this project was comprised of clinicians, academics and people living with MS. The researchers leading this review had expertise in qualitative research methods and a variety of review methodologies.

### 2.1 Research question

The overarching question underpinning this review was as follows: *How do people experience living with MS?* Two further questions were defined: (a) What are the key experiences explored in the qualitative literature? and (b) What common themes underpin these experiences?

### 2.2 Searches

Systematic searches were conducted in ProQuest, PubMed, CINAHL and PsychINFO using the search string (‘multiple sclerosis’) AND (experienc* OR perception* OR perspective* OR attitude* OR belief* OR value* OR view*) AND (qualitative OR ‘focus group*’ OR interview* OR narrative*).

### 2.3 Study inclusion and exclusion criteria

Inclusion criteria were studies with empirical qualitative data about adults’ subjective experiences of living with MS (2010 to January 2019). Mixed‐method studies were included if qualitative data could be extracted. Studies that focused on the experience of others (eg carer/family/health‐care professionals) were excluded. Studies in the grey literature and those not written in English were also excluded.

Experiences of the person with MS included physical, social and/or psychological impacts of the disease, health systems and services, health‐care professional interactions and disease management. Studies describing experiences related to specific interventions or treatments (eg a specific activity programme as opposed to all physical exercise or a specific drug as opposed to all disease‐modifying therapies) were excluded.

### 2.4 Study quality assessment

All included studies were appraised using the Critical Appraisal Skills Program (CASP) qualitative checklist^25^ by two researchers working independently. Title and abstract, and full‐text screening was performed by two reviewers. Any disagreements were resolved by a third reviewer.

### 2.5 Charting the data

A thematic analytical approach was adopted to provide a rich description of MS experiences.^26^ Data familiarization was achieved through several stages of article review. Coding and interpretation began at title and abstract screening, and were refined as the data were reviewed. Initial coding involved arranging‐related types of experiences conceptually into categories, capturing disease domains (diagnosis, progression and relapse, physical and psychological symptoms) and contexts of people's lives (work, social and family life; the health sector). We coded and compared the breadth and commonalities of experience across these domains and contexts. Final coding was conducted using NVivo 12, a qualitative data analysis computer software package.^27^

We undertook blinded audits to ensure consistency of codes and concepts between reviewers. Any differences in approaches were resolved through discussion across the research team.

### 2.6 Synthesis with knowledge experts

To improve the authenticity of the synthesis,^28^ research team members with MS read the analyses and contributed personalized reflections, which were translated into I‐poem^29^ or narratives to capture the complex subjectivities of persons with lived experience responding to research on lived experience of the same disease.

### 2.7 Ethical approval

This review did not include direct involvement with human participants; it was a secondary analysis of research data, and therefore in accordance with the National Statement on Ethical Conduct in Human Research 2007 (Updated 2018) did not require ethical approval.^30^

## 3 RESULTS

**FIGURE 1:**
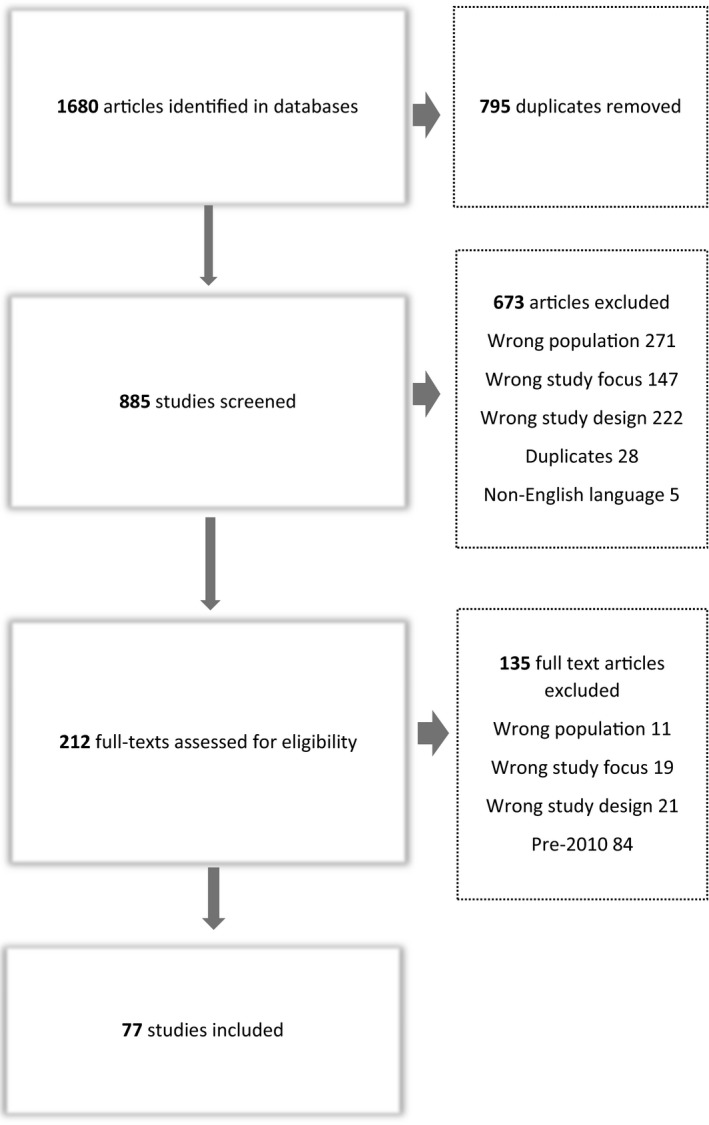
Search flow diagram

Of 1680 articles identified in the initial search, 77 met the inclusion criteria (Figure 1). Ages of participants ranged from 18 to 81 years; two‐thirds were female. The data collection method used most frequently was interviews (84%), followed by focus groups (14%) (Table 1). The country of participants’ origin most represented in the studies was the UK (23%), followed by the United States (17%); Scandinavian countries (12%); and Iran (12%).

**TABLE 1:** Included studies: Descriptors and key included domains/contexts

Author (Abbr)	Year	Country	Population	n	♀	♂	Age	Aims	Key findings	P1	P2	P3	P4	P5
Adamson et al	2018	USA	PwMS	14	13	1	27‐70	To understand ways that individuals with MS who had a recent relapse describe the roles of physical activity regarding MS itself, relapse, and disability identity	There is both empowerment and guilt in physical activity. Empowerment comes from feelings of taking control of MS, and guilt may develop through perceptions of disengaging with exercise			√	√	
Al‐Sharman et al	2018	Jordan	PwMS	16	8	8	22‐57	To explore experiences and challenges of living with MS from a Jordanian perspective	Provides an overview of the experience of living with MS in Jordan, as conceptualized through two distinct areas of experience – that is, disease related experiences and experiences with the health‐care system	√	√	√	√	√
Aminian et al	2017	Canada	PwMS	15	12	3	23‐61	To see whether replacing sedentary behaviour with light activities to manage MS symptoms	Adults with MS were open to replace sitting with light activities		√	√		√
Anderson et al	2013	UK	Women with MS	9	9		18‐50	To identify concerns with pregnancy and mothering	Women with MS have difficulty in finding the correct information on how pregnancy will affect their MS. Main concerns surround theirs and their baby's future well‐being		√	√		√
Asanao et al	2015	Canada/USA	PwMS relapse	17	16	1	26‐69	To explore how PwMS process their relapse experience and manage the consequences	There is a need for multidisciplinary post‐relapse care beyond restoring functional limitations in the acute phase of relapse				√	
Blundell Jones et al	2014	UK	Women with MS	10	10		30‐64	To explore the emotional experiences and help‐seeking behaviours of women with MS	Non‐help seeking was influenced by desire to keep things normal and a lack of knowledge regarding service provision. More holistic care from services was desired	√	√	√	√	
Bogenscutz et al	2016	USA	PwMS	27	19	6	20‐69	To examine work‐related experiences of PwMS	Unpredictability of MS, effects on cognitive capabilities and physical stamina, and concerns about seeking workplace accommodations severely undermined prospects for continued work and education					√
Bogosian et al	2017	UK	PwMS	34	25	9	41‐77	To examine cognitive and behavioural challenges and adaptations for PwMS	Adjusting to MS following diagnosis a fluid process and involves decisions about whether to reveal or conceal the condition	√	√	√	√	
Browne et al	2015	UK (Ireland)	PwMS	19	11	8	37‐64	To understand how bladder dysfunction interferes with quality of life	Bladder dysfunction is a major disruption to living with MS. In view of difficult to navigate health systems and services, many people with MS attempt to self‐manage	√	√	√		√
Brunn Helland et al	2015	Norway	PwMS	27	16	11	37‐71	To identify factors influencing use of rehab services	Communication skills including information giving skills of neurologist on diagnosis need improvement, and patients need equal access to information about rehabilitation options	√	√	√		√
Chard	2017	USA	PwMS doing aquatic exercise	45			18+	To determine attitudes and experiences of PwMS re aquatic exercise	Both MS‐specific exercise groups and general exercise groups provide positive exercise experiences, a history of previous exercise is not key to taking it up, class satisfaction based of sense of acceptance and good instructor, and HCPs could play a stronger role in encouraging PwMS		√	√	√	√
Coenen et al	2011	Germany	PwMS	27	19	8	28‐73	To explore impacts of MS on functioning and disability	Functioning and disability in MS can be influenced by a range of complex and multidimensional environmental and personal factors		√	√		
Cowan et al	2018	Australia	PwMS after discharge from rehabilitation	15	9	6	25‐64	To explore lived experiences after inpatient rehabilitation and discharge home	Physical and mental fatigue impacted on all aspects of day‐to‐day life after rehabilitation. A desire for independence and concerns over burden on family were experienced, as was a loss of valued roles including work			√	√	√
de Ceuninck van Capelle et al	2016	Netherlands	PwMS recently diagnosed	10	8	2	27‐51	To understand how recently diagnosed PwMS experience family life	MS affected family life and perceived ability to care for their family and home. Given the pivotal role of this worry, more family‐centred care should be integrated into MS care			√		√
de Ceuninck van Capelle et al	2017	Netherlands	PwRRMS	10	8	2	27‐51	To explore patient's perspectives on using disease‐modifying therapies (DMTs) for MS	The use of DMTs and dealing with advice to start them are a complicated treatment step. Decision is not made in isolation, but is grounded in the support/advice from relatives and friends	√	√	√	√	√
Deghan‐Nayeri et al	2018	Iran	Women with MS	25	25		21‐45	To understand the sexual life and experiences of Iranian women with MS in an Iranian cultural context	Hiding sexual problems from husbands is common and sexual awareness and education should be extended in the rehabilitation team		√	√		
Deghan‐Nayeri et al	2017	Iran	PwMS	11	6	5	24‐46	To understand factors affecting how PwMS cope	Coping with MS is complex and affected by both individual and broader factors, including social and economic conditions			√	√	
Deghan‐Nayeri et al	2018	Iran	PwMS	11	6	5	24‐46	To understand the features of coping with MS	Identified four key features of coping with MS: acceptance, relationships, self‐regulation and self‐efficacy			√		
Dennison et al	2011	UK	PwMS	30	22	8	40‐50	To identify the adjustment required when diagnosed with MS	Services for people with early‐stage MS need careful attention to ensure they are sensitive and supportive rather than threatening and alienating	√		√		√
Dennison et al	2016	UK	PwMS	15	12	3	31‐68	To explore how pwMS experience prognostic uncertainty and communication with HCPs	PwMS developed beliefs and expectations about their prognosis, particularly about pace of worsening, with minimal input from HCPs. Prognostic information threatened a need to remain present focused and was considered emotionally dangerous	√	√	√	√	
Dlugonski et al	2012	USA	Women with MS	11	11		18‐64	To better understand the adoption and maintenance of physical activity in women w MS	Consideration of physical activity beliefs, motivations and strategies may be useful in designing behavioural interventions to increase physical activity			√		√
Encarnação et al	2016	Portugal	PwMS	15	9	6	31‐60	To understand the perception of faith in PwMS	Faith as a resource can achieve a positive outcome and assist PwMS to develop hope			√		√
Fallahi et al	2014	Iran	PwMS	25	18	7	20‐55	To explore the experiences of PwMS in confronting their diagnosis	Confronting a diagnosis of MS may involve a need for information, decisions around revealing a diagnosis, faith in god and emotional reactions including denial, anxiety fear and confusion	√	√			√
Frost et al	2017	UK	PwPMS	14	10	4	40‐67	To explore experiences of diagnosis and self‐management	Gender differences with coping and living with MS were identified. These are more apparent in early stages and at time of diagnosis	√	√	√	√	√
Gaskill et al	2011	USA	PwMS who are experiencing suicidal ideation (SI)	16	11	5	21‐64	To determine whether SI is greater in PwMS than the general population	Perceived loss of control was highlighted by all participants as contributing to SI. Interventions that seek to increase control in other areas of people's lives could serve as a buffer to SI			√		
Ghafari et al	2014	Iran	PwMS who are married	25	18	7	20‐55	To determine the extent and type of spousal support	PwMS would rather have more emotional support than physical support					√
Ghafari et al	2015	Iran	PwMS	25	18	7	20‐55	To identify themes and subthemes of pwMS in relation to their hospital experiences	Main themes identified were religiosity, information seeking, seeking support, hope rearing, emotional reactions, concealing disease, fighting disease and disability		√			
Giovannetti et al	2017	Italy	PwMS who have requested psychosocial support	19	13	6	19‐57	To explore adjustment to MS	Psychosocial interventions can support patients to adjust and accept diagnosis of MS	√		√		
Harrison et al	2015	UK	PwMS who have major pain issues	25	19	6	18‐70	To explore PwMS experiences and responses to pain, and their perspectives of pain management	Identified pain‐related beliefs, emotional reactions and disparate pain management attitudes			√		√
Hosseini et al	2016	Iran	PwMS	34	25	9	23‐54	To identify the nature of leisure activities of PwMS in the context of Iranian culture	Six categories physical, social, individual, art/cultural, educational, and spiritual/religious. Useful to understand for mental health promotion purposes and tailored interventions			√		
Hunt et al	2014	UK	PwMS in Ireland	5	3	2	40‐65	To explore meanings of leisure‐based visual art making for PwMS	PwMS valued creative art making, developed friendships and it enabled respite from worry			√		√
Kayes et al	2011	Australia	PwMS	10	7	3	34‐53	To explore barriers to physical activity	Barriers to physical activity are complex due to variability of MS symptoms		√	√	√	
Kirk‐Brown and Van Dijk	2014	Australia/NZ	Employed PwMS	40	28	12	18‐65	To examine what psychosocial support PwMS require post disclosure to maintain employment	Management responses to disclosure should focus on abilities and inclusive decision making					√
Knaster et al	2011	USA	PwMS	12	8	4	41‐71	To examine how PwMS self‐manage	Self‐management involved mainlining control and adapting and altering to capabilities to perform valued roles			√	√	
Lee Mortensen & Rasmussen	2017	Denmark	PwMS	40	29	11	18‐63	To explore the main factors affecting patients' preferences regarding MS treatment and health care	Ability to uphold meaningful role functioning was crucial to treatment priorities. Unmet information and support needs from HCPs especially at time of diagnosis	√	√	√		
Lexell et al	2011	Sweden	PwMS	10	6	4	41‐67	To understand how PwMS adapt to their changed physical circumstances	Participants had to be prepared to adapt to rapidly changing circumstances on a daily basis. This process was on‐going and dynamic, but motivated through achieving a desired self or family life			√		√
Lohne et al	2010	Norway	PwMS	14	8	6	39‐66	To explore how PwMS experience and understand dignity in the context of a rehabilitation ward	Invisibility of MS symptoms may influence an experience of self as invisible, and the perception that needs are not respected, affecting dignity		√	√		
Lynass and Gillon	2017	UK (Scotland)	PwMS	5	3	2	18+	To explore the experience of person‐centred counselling for PwMS	Counselling was found to be helpful. Empathy and non‐directive and non‐judgemental approaches were valued as were counsellor's knowledge of MS			√		
Lynd et al	2018	Canada	PwMS	23	18	5	20‐72	To explore patient preferences regarding drug treatments	Patients consider the impact and likelihood of benefits and side‐effects when making drug treatment decisions		√	√		
Maghsoodi & Mohammadi	2018	Iran	Women with MS	10	10		30‐62	To explore the process of restoring social esteem to women with MS in Iranian culture	Social esteem was severely affected by sense of abandonment, rejection from family and friends, financial problems and feeling a burden	√	√			√
Masoudi et al	2015	Iran	PwMS	23			20‐50	To identify experience of continuity of care for PwMS in Iran	Patients requested need for dignity and respect from carer givers, as well as empathy and knowledge of MS		√	√		
Meade et al	2016	USA	PwMS	74			20‐81	To determine the benefits/quality outcomes of working for PwMS	Participants reported a range of motivations to work including compensation, personal well‐being and to help others		√	√		√
Moriya & Kutsumi	2010	Japan	PwMS	9	6	3	31‐57	To explore the impacts of fatigue in PwMS, especially in relation to social life and interpersonal relations	Fatigue has far reaching physical, psychological and social implications for PwMS		√	√		√
Moriya & Suzuki	2011	Japan	PwMS	17	13	4	20‐59	To ascertain differences in symptoms experienced by individuals with MS per disease severity	Characteristics of experiences may differ because of disease severity	√		√		√
Morley et al	2013	UK	PwMS experiencing spasticity	10	7	3	20‐69	To investigate the impact of spasticity on the lives of PwMS	Spasticity has physical, psychological and social consequences for people with MS		√	√		√
Mozo‐Dutton et al	2012	UK	PwMS	12	8	4	34‐71	To explore the impact of MS on perceptions of self	The physical body is intrinsically linked with sense of self; however, the onset of MS does not necessarily equate to a loss of self	√	√	√		√
Newland et al	2012	USA	PwMS who discuss symptoms	16	12	4	25‐58	To characterize the symptoms of PwMS in their own words	Certain common symptoms may be characterized by as association with other MS symptoms. This study found a need to develop a clinical tool to document changes in symptoms			√		
Olsson et al	2010	Sweden	Women with secondary progressive MS	15	15		35‐70	To describe the meanings of feeling well for women with MS	Feeling well in women with MS influenced by finding a pace where 'daily life goes on' despite living with illness			√		√
Olsson et al	2011	Sweden	Women with secondary progressive MS	15	15		35‐71	To understand the meanings of being received and met by others as experienced a woman with MS	Women feel valued when accepted as 'normal' and disappointed/not valued when viewed as abnormal and constantly needing to justify their situation		√			√
Parton et al	2018	Australia	Mothers with MS	20	20		26‐54	To examine how women with MS construct and experience motherhood	Complexity of mothering with MS highlighted as women negotiate the fear of being a bad mother, as constructed by perceptions of self‐sacrifice and meeting their children's needs, with building resilience and character in their children. MS was a catalyst for some to engage in self‐care and provided a buffer from guilt	√		√	√	√
Parton et al	2017	Australia	Mothers with MS	20	20		26‐55	To understand how women with MS construct their sense of self as a mother	Women with MS identified negative and positive aspects of sense of self as a mother. Health professionals can assist women better knowing how they experience living with MS as a mother					√
Payne & Kathryn	2010	NZ	Mothers with MS	9	9		22‐45	To explore experience of mothers with MS, and elicit the strategies used to manage mothering and MS	Support is pivotal to mothers with MS, as is the need to conserve energy to manage fatigue		√	√		√
Ploughman et al	2012	Canada	Older PwMS	18	14	4	56‐81	To explore older people's experiences of ageing with MS	Dealing with loss and navigating barriers, especially in the areas of employment, independence and social participation are critical components of self‐management'	√	√	√	√	√
Plow & Finnlayson	2012	USA	PwMS	8	6	2	29‐58	To explore the experience of how PwMS participate in domestic life activities	Nutrition plays an important yet overlooked part in MS management – difficult symptoms, the social environment and a lack of information play a role in preventing PwMS from engaging in healthy eating behaviours		√	√		√
Pretorius & Joubert	2014	South Africa	PwMS	10	7	3	38‐71	To explore the experiences of PwMS in the South African (SA) Context	The study highlights several key challenges (diagnosis, daily life, invisible illness and medical aid) and resources (social support, mobility aids, religion and knowledge) for PwMS in SA	√	√	√		√
Riazi et al	2012	UK	PwMS in care homes	21	10	11	43‐80	To examine the experiences of care home residents with MS	Quality of life in care home residents could be improved by involving family, supporting transitions and improving access to services such as rehabilitation		√			√
Rintel et al	2012	USA	PwMS who had received mental health care	54	44	10	18+	To explore the experience of mental health care in PwMS	Mental health care should be provided upon diagnosis of MS, and providers should be familiar with MS	√	√	√		
Russell et al	2018	Australia	PwMS with recent diagnosis	11	9	2	31‐70	To explore responses to diet after recent diagnosis of MS	Lack of information specific to MS, and specific to individuals with MS, surrounding dietary advice	√	√	√		
Senders et al	2016	USA	PwMS	34	30	4	18+	To further understand how stress is addressed in the MS medical visit	Psychological stress in PwMS is not adequately addressed during medical visits		√			√
Sharifi & Abbaszadeh	2016	Iran	PwMS	13	6	7	28‐51	To explore the daily social interactions that affect the dignity of PwMS	A range of personal and social factors can affect perceived dignity of PwMS. Dignity can be promoted through moderation of dignity‐threatening factors, and improvement of dignity enhancing factors					√
Skar et al	2014	Norway	PwMS who recently completed rehabilitation	10	6	4	45‐61	To explore the experience of rehabilitation and how it might provide psychosocial benefits	Inpatient rehab instilled sense of community, recognition and empowerment in an environment where PwMS felt free from stigma			√		√
Skovgaard et al	2014	Denmark	PwMS	11	11		31‐39	To explore how people with MS consider the risks of combining conventional and complementary medicines (CAM)	PwMS considered CAM to be safe as guided by the 'naturalness' of treatments, their own body sensations, trust in their CAM practitioner and a lack of dialogue from their medical doctor		√			
Skovgaard et al	2014	Denmark	PwMS	17	15	2	18+	To explore issues surrounding exclusive CAM use in pwMS	Use of exclusive CAM associated with beliefs and experiences of avoiding chemical substances, strengthening the body, increasing controls and participation in one's health, and maintaining body sensations which were seen as valuable in guiding treatments decisions		√	√		
Smith et al	2015	NZ	Men with MS	18		18	36‐68	To examine fatigue and exercise experience of men with MS	Fatigue has physical and psychological consequences for men, but goal readjustment aids men to stay engaged in exercise			√		√
Smith et al	2011	NZ	PwMS who engage in community‐based exercise	10	10		28‐70	To explore how PwMS experience fatigue and how this influences participation in community‐based exercise	MS‐related fatigue is unpredictable and controlling. Regaining control over fatigue is a complex process influenced by multiple factors including feeling supported, managing limits and individual wellness philosophies/goals		√	√		√
Sosnowy	2014	USA	Women with MS	9	9		18+	To examine the experiences and perspectives of women who blog about their MS	Blogging provides an opportunity to gain information and resist dominant medical discourses		√			
Soundy et al	2012	UK	PwMS involved in rehabilitation	11	7	4	42‐69	To understand how PwMS in a rehabilitation setting express hope	Despite acceptance of loss, meaning and values in their life, PwMS could defy their illness through maintaining hope and a sense of purpose in life. Physiotherapists need to support this process during rehabilitation			√	√	√
Stennett et al	2018	UK	Community dwelling PwMS	16	12	4	47‐72	To explore the meaning of physical activity to people with MS who live in the community	PwMS may describe a broad, multidimensional concept of physical activity that reflects social engagement, uncertain trajectories and coping with their illness			√		√
Stern & Goverover	2018	USA	Men with MS	3		3	50‐57	To present perspectives of everyday technology use for men with MS	Facilitating everyday technology use in men with MS may promote health and quality of life	√				√
Stone et al	2013	Canada	PwMS working in academia	35	20	10	33‐72	To explore academics with MS experiences of seeking employment accommodations	Academics with MS who seek workplace adjustments can be conceptualized in terms of needing to 'go through the back door' – concealing disabilities to avoid stigma			√		√
Strickland et al	2017	UK (Scotland)	Recently diagnosed PwMS	10	8	2	25‐45	To understand the impact of a diagnosis of MS	Diagnosis of MS results in a separation from the pre‐symptomatic self, to an evolving reconstruction of identity influenced by social roles, uncertainty, availability of health care	√	√			√
Tabuteau‐Harrison et al	2016	UK	PwMS	15	11	4	42‐67	To determine whether adjustment to MS is determined by social group factors	Social groups play an important role in adjusting to MS, and in continuing valued roles and relationships			√		√
Turpin et al	2018	Australia	PwMS who experienced fatigue	13	11	2	25‐67	To determine how individuals experienced MS fatigue	Fatigue is a challenging and debilitating MS symptom which is poorly understood and largely invisible to others			√		√
van der Meide et al	2018	Netherlands	PwMS	13	13		18+	Examines the bodily experiences of PwMS	People with MS experience the body through oscillating dimensions of bodily uncertainty, having a precious body, being a different body and the mindful body			√	√	√
Vijayasingham et al	2017	Malaysia	PwMS	10	6	4	25‐46	To describe how PwMS perceive and negotiate the long‐term course of their employment	Holistic life management decisions contribute to on‐going but also disrupted work trajectories	√		√	√	√
Willson et al	2018	Italy	Mothers with MS	16	16		N/A	To explore the perceived influence of MS on mothers in an Italian socio‐cultural context	MS can affect ability to participate in mothering tasks and cause subsequent feelings of difference and loss, influenced by a desire to stay in control and perceptions of stigma, which impact on women's identity as mothers	√		√	√	√
Yilmaz et al	2017	Turkey	Women with MS	21	21		23‐51	Explores the impacts of MS in women on sexual, physical and emotional functioning	MS influences uncertainty in terms of illness and marriage, affects sexuality and influences a perceived inadequacy to engage in the role of wife and mother. Women felt a lack of support and acknowledgement of the impacts of MS on their sexual lives			√	√	√

Note

Domains and contexts: P1: Experiences of receiving the diagnosis; P2: Experiences of health services and health professionals; P3: Experience of managing physical and psychological symptoms; P4: Experience of disease progression and relapse; P5: Experiences and effects on work, social and family life.

Abbreviations: HCP, health care provider; MS, multiple sclerosis; PwMS, people with MS.

### 3.1 Quality assessment

The quality of all 77 studies was considered acceptable using the CASP tool (Table S1). The criterion of adequate consideration of the relationship between researcher and participants was met in only 34% of studies. 12% of studies used recruitment strategies which we did not consider appropriate to address the aims of their study. In 13% of studies, ethical issues were either inadequately addressed, or information about consent, recruitment and/or obtaining approval of a human research ethics committee was not provided.

### 3.2 Qualitative synthesis

We identified five overarching themes describing people's experiences of living with MS: (a) the quest for knowledge, expertise and understanding, (b) uncertain trajectories (c) loss of valued roles and activities, and the threat of a changing identity, (d) managing fatigue and its impacts on life and relationships, and (e) adapting to life with MS. (Table 2).

**TABLE 2:** Thematic framework

Theme	Domain or context	Experiences
A quest for knowledge, expertise and understanding	Receiving the diagnosis	Insufficient information and support from health‐care professionals ^15^, ^31^, ^33^, ^35^, ^37^, ^38^, ^39^ Relieved to have a diagnosis ^29^, ^30^, ^31^, ^32^ and a sense of validation ^11^, ^29^, ^32^, ^33^ Positive experience of being directed to support services ^32^, ^33^
	Physical and psychological symptoms	Extensive self‐directed information seeking ^8^, ^15^, ^30^, ^31^, ^34^, ^35^, ^36^, ^37^ Difficulties in accessing the information needed to manage day‐to‐day impacts – physical ^9^, ^33^, ^41^, ^42^, ^52^, ^53^, ^100^ and psychological ^9^, ^33^, ^41^, ^42^, ^53^ symptoms, and prevent future relapses ^9^, ^33^, ^41^
	Work, social and family life	Insufficient information to make important life decisions, such as having children ^9^, ^33^, ^41^ Feeling misunderstood at work,^16^, ^18^, ^20^, ^31^, ^33^, ^43^, ^46^ in social situations ^16^, ^18^, ^20^, ^31^, ^33^, ^43^, ^46^, ^47^, ^48^ and in family life ^18^, ^20^, ^33^, ^43^, ^46^, ^47^, ^48^ Preconceived ideas about MS and what a future living with MS might look like ^47^ Managing others’ perceptions of MS (invisible symptoms, stigma and justifying illness impacts) ^12^, ^18^, ^20^, ^31^, ^32^, ^33^, ^46^, ^48^, ^49^, ^50^ Peer support – receiving respite,^42^ motivation,^16^, ^40^, ^47^ information ^8^, ^31^, ^40^, ^48^, ^53^, ^82^ and understanding ^8^, ^31^, ^40^, ^47^, ^48^, ^50^, ^53^, ^82^ from other people living with MS Avoiding peer support – not wanting to be defined by illness or disability; avoiding reminders of the threat of potential future disability ^8^, ^15^, ^48^, ^64^
	Health services and health professionals	Lack of personally tailored and specific information from health‐care providers (generic advice/knowledge) ^9^, ^29^, ^35^, ^38^, ^40^, ^41^, ^42^, ^43^, ^44^ Unknown/uncertain impacts from treatments and interventions ^13^, ^17^, ^37^ Lack of referral to support services (including lifestyle and psychological interventions) ^32^, ^33^, ^38^, ^40^, ^41^, ^53^ Receiving advice on diet and physical activity that was not clear or relevant ^37^, ^47^ Variable information related to having children – from supportive to discouraging ^9^, ^10^ Clinicians’ focus (eg pharmacological/focus on one body system) ^31^, ^33^, ^34^, ^41^, ^46^, ^50^ Clinicians’ communication style and lack of time and opportunity to discuss prognosis ^35^, ^46^ Alternative paths of self‐directed research using resources such as the internet,^9^, ^18^, ^31^, ^35^, ^52^, ^53^, ^54^, ^55^ books,^9^, ^18^, ^31^, ^35^, ^54^ peer group support networks,^9^, ^18^, ^31^ media,^18^, ^52^ friends,^52^, ^53^ family,^54^ spiritual leader ^54^ and MS associations ^35^, ^53^, ^55^
Uncertain trajectories	Prior to diagnosis	Uncertainty prior to diagnosis in relation to long‐standing and unsettling symptoms, for which there was no known cause ^29^, ^36^, ^41^, ^49^, ^67^
	At diagnosis/health services and health professionals/disease progression and relapse/physical and psychological symptoms	Uncertainty of future progression, recovery and clinical course, including what future symptoms might be experienced and how disabling they might be ^7^, ^8^, ^11^, ^19^, ^29^, ^33^, ^35^, ^41^, ^45^, ^48^, ^51^, ^57^, ^61^, ^67^, ^101^, ^102^, ^103^ Fear of potential futures, including cognitive and vision decline,^7^, ^61^ loss of mobility, and fear of a potential need for aids, especially a wheelchair ^7^, ^8^, ^11^, ^45^, ^51^, ^102^
	Work, social and family life	Concern about the ability to continue to work, maintain independence and provide for themselves and their family ^33^, ^41^, ^51^, ^54^, ^57^, ^63^ Fears of passing MS onto children and worry about the impact that MS might have on future ability to parent ^9^, ^11^, ^12^, ^33^, ^41^, ^67^ Concerns about being a care burden on family and significant others in the future were also expressed ^11^, ^12^, ^33^, ^43^, ^63^, ^66^, ^68^, ^78^, ^104^ Starting a family (information, support) ^9^, ^10^
Loss of valued roles and the threat of a changing identity	Physical and psychological symptoms	Valued activities included parenting,^12^, ^33^, ^48^, ^51^, ^70^, ^78^ playing with children,^51^, ^70^, ^102^ work,^8^, ^30^, ^32^, ^33^, ^41^, ^48^, ^51^, ^57^, ^73^, ^76^, ^102^, social activities,^16^, ^30^, ^32^, ^41^, ^48^, ^51^, ^57^, ^64^, ^72^ activities of daily living,^8^, ^12^, ^16^, ^30^, ^51^, ^64^, ^67^, ^70^, ^75^, ^78^, ^103^, and physical activity,^8^, ^30^, ^32^, ^41^, ^45^, ^48^, ^51^, ^57^, ^58^, ^70^, ^72^, ^76^, ^102^, ^103^ Personal attributes perceived as under threat from MS included health,^19^, ^33^, ^51^, ^57^, ^70^ independence,^8^, ^51^, ^64^, ^70^ strength,^57^, ^76^ masculinity,^65^, ^76^ intellect,^39^, ^80^ youth ^78^ and physical appearance ^41^
	Work, social and family life	Valued roles included those of worker,^32^, ^33^, ^39^, ^57^ provider,^32^, ^57^ professional,^32^, ^73^ parent,^33^, ^102^ partner or spouse ^32^, ^33^ Changing roles and abilities affecting family dynamics ^12^, ^30^, ^48^, ^63^ Inability to provide for family through loss of employment and income ^33^, ^61^, ^64^, ^65^, ^66^, ^67^, ^68^, ^69^ Physical and cognitive barriers to continuing work ^48^, ^51^, ^55^, ^57^, ^71^ Concealing MS diagnosis: work ^7^, ^30^, ^33^, ^57^, ^64^, ^73^ and social life ^7^, ^30^, ^33^, ^64^ reasons for – professional perceptions,^64^, ^73^ not wanting to be seen as different or disabled,^33^, ^40^, ^45^, ^57^, ^63^, ^64^, ^73^ uncertainty about how others would respond,^7^, ^45^, ^57^, ^71^ and wanting to avoid unnecessary pity,^7^, ^33^, ^71^, ^102^, attention ^40^, ^45^ and stigma,^7^, ^40^, ^71^, ^102^ Disclosing MS diagnosis: being supported and accommodated,^64^, ^73^, ^74^ and negative reactions, including having their work competency questioned,^41^, ^57^, ^74^ being treated dismissively,^57^, ^73^, ^74^ being overlooked for promotion ^76^ and abandoned by friends ^47^
Managing fatigue, and its impacts on life and relationships	Physical and psychological symptoms	Fatigue was described as a symptom that featured across multiple contexts of people's lives ^10^, ^11^, ^12^, ^14^, ^16^, ^18^, ^20^, ^29^, ^32^, ^33^, ^39^, ^41^, ^45^, ^47^, ^48^, ^49^, ^51^, ^55^, ^58^, ^59^, ^64^, ^70^, ^72^, ^75^, ^76^, ^77^, ^78^ Preceding diagnosis ^18^, ^31^, ^49^, ^67^ and, later, as a flag indicating relapse or progression ^20^, ^30^, ^47^ A wide range of related physical and psychological symptoms included depression and anxiety,^18^ cognitive and mood changes,^43^ pain,^58^ bladder and bowel dysfunction,^77^ spasms,^60^ mobility problems and paralysis,^20^, ^55^ numbness, pins and needles,^38^ trigeminal neuralgia ^32^ and optic neuritis.^33^ Fatigue was associated with heat sensitivity and vice versa, and had considerable impacts on people's lives ^33^ Different descriptions of fatigue were used synonymously; these included being ‘washed out’,^65^ ‘shut down’,^61^ ‘really heavy’,^16^ ‘empty’,^78^ ‘running out of batteries’,^20^, ^49^ as well as descriptions such as ‘all consuming’,^49^ ‘a blanket’,^60^ ‘stuck to the skin’ ^22^ and like ‘wearing a trench coat […] made of lead’ ^78^ Effective management strategies: effective use of exercise ^14^, ^16^, ^47^, ^76^ and diet ^49^ incorrect use of exercise causing fatigue ^14^, ^47^ lack of information about how to manage fatigue ^18^, ^20^
	Work, social and family life	Impacts – work,^18^, ^49^, ^51^, ^70^, ^75^, ^76^ social life,^18^, ^45^, ^49^, ^51^, ^75^, ^76^, ^77^ family life ^12^, ^18^, ^45^, ^49^, ^51^, ^52^, ^75^, ^76^, ^78^ everyday activities, physical and psychological health ^14^, ^16^, ^47^, ^76^ Perceived lack of legitimacy ^38^, ^40^ repeatedly having to explain or justify limitations to friends, family and workplaces ^31^, ^40^, ^46^, ^50^
Adapting to life with MS	Physical and psychological symptoms	Technology enabled: connection to community (internet) ^82^ participation in online exercise programs ^43^ monitoring activity and fitness levels (Fitbits) ^18^ assistance with activities of daily living activities (mobile phone) ^53^ and work‐related tasks (iPad and iPod) ^59^ Pacing, planning and ensuring time to rest,^11^, ^39^, ^55^, ^104^ Changing exercise routines (eg choosing swimming due to heat intolerance),^53^, ^103^
	Work, social and family life	Specific coping methods included staying in the present and shifting focus away from MS,^10^, ^33^, ^35^, ^48^, ^70^, ^102^, ^104^, concealing the diagnosis,^63^, ^64^, ^71^ altering self‐expectations, recognizing limits and adapting tasks,^11^, ^33^, ^47^, ^55^, ^63^, ^64^, ^76^, ^79^, ^82^, ^103^, ^104^ Drawing on personal resources: work ^64^, ^69^ spiritual faith ^33^, ^70^, ^81^ family support ^10^, ^31^, ^82^ (including financial support to access care ^13^, ^66^) social interaction, including engaging with others with MS ^8^, ^16^, ^40^, ^47^, ^48^, ^82^ finding and maintaining a sense of purpose,^48^, ^69^ and engaging in physical activity ^16^, ^53^, ^72^, ^102^
	Health services and health professionals	Support through rehabilitation,^48^, ^51^, ^75^ counselling ^62^, ^75^ and pharmacological interventions ^41^ important The importance of health services was most apparent when access difficulties were experienced ^6^, ^31^, ^46^, ^54^, ^66^, ^71^

#### 3.2.1 A quest for knowledge, expertise and understanding

This theme included experiences related to diagnosis, treatment, and information and support seeking. While some people described a sense of relief^29^, ^30^, ^31^, ^32^ and validation^11^, ^29^, ^32^, ^33^ at diagnosis, followed by direction to support services,^32^, ^33^ many highlighted extensive self‐directed efforts to meet their information needs at an already stressful time.^8^, ^15^, ^30^, ^31^, ^34^, ^35^, ^36^, ^37^ Several studies referred to people's experiences of receiving insufficient information and support from health‐care professionals at this time.^15^, ^31^, ^33^, ^35^, ^37^, ^38^, ^39^ People described the provision of generic advice from health‐care providers, rather than personally tailored and specific advice.^9^, ^29^, ^35^, ^38^, ^40^, ^41^, ^42^, ^43^, ^44^ In two studies, women described receiving inadequate and conflicting information related to having children – from supportive to discouraging.^9^, ^10^

People with MS often had to navigate their own preconceived ideas about MS and what their future living with MS might look like; for example, they may have inferred from the frequent image of someone in a wheelchair used in popular representations of MS that this would be the outcome for all with this diagnosis.^47^ Overall, a general lack of information and knowledge about MS in the community extended to their experiences of being misunderstood at work,^16^, ^18^, ^20^, ^31^, ^33^, ^43^, ^46^ in social situations^16^, ^18^, ^20^, ^31^, ^33^, ^43^, ^46^, ^47^, ^48^ and in family life.^18^, ^20^, ^33^, ^43^, ^46^, ^47^, ^48^ Their MS symptoms were often referred to as ‘invisible’, obliging them to assert the impacts of MS on daily life and to help others understand a hidden disability.^12^, ^18^, ^20^, ^31^, ^32^, ^33^, ^46^, ^48^, ^49^, ^50^ Conversely, while information was welcomed by most, some described being bombarded,^15^, ^51^ inundated,^54^ and overwhelmed^38^ by advice and disease details. In response, some chose to manage anxiety about the future by only researching those symptoms that were of current concern to them.^38^ People with MS described varied alternative paths of self‐directed research using resources such as the internet,^9^, ^18^, ^31^, ^35^, ^52^, ^53^, ^54^, ^55^ books,^9^, ^18^, ^31^, ^35^, ^54^ peer group support networks,^9^, ^18^, ^31^ media,^18^, ^52^ friends^52^, ^53^ and MS associations.^35^, ^53^, ^55^

#### 3.2.2 Uncertain trajectories and a need to plan

This theme described the inherent uncertainty people with MS experienced across all aspects of their lives. It was expressed around the time of diagnosis,^7^, ^8^, ^10^, ^15^, ^29^, ^30^, ^31^, ^32^ regarding treatment,^6^, ^13^, ^39^, ^42^, ^56^ and in terms of the potential future impacts of MS progression, especially on work,^33^, ^41^, ^54^, ^57^ family and relationships.^9^, ^12^, ^19^, ^45^

Several studies included experiences of physical and psychological symptoms presenting themselves acutely and without warning. These included bladder symptoms,^44^ pain,^58^ fatigue,^32^, ^49^, ^58^, ^59^ spasticity,^62^ speech problems,^63^ balance disturbances,^43^ and cognitive and mood changes.^64^ This unpredictability caused worry,^41^, ^42^, ^51^ made it difficult to plan,^36^, ^41^, ^42^, ^51^, ^56^, ^58^ and disrupted valued roles and activities,^20^, ^36^, ^41^, ^42^, ^51^, ^56^, ^58^, ^60^ and everyday routines.^20^, ^41^, ^42^, ^60^

Lack of certainty about treatment effectiveness, including impact on clinical course, made it difficult to make decisions about which treatments to choose, especially considering potential significant side‐effects and impact on quality of life.^13^, ^39^ In a study of the exclusive use of alternative medicine by people with MS, interviewees described the lack of certainty regarding the impact and long‐term effects of conventional medicines as a deterrent to their use, leading to the adoption of alternative modes of therapy, which were represented as delivering more certainty of outcome.^19^ At the same time, uncertainty surrounding clinical course, and prognostication, appeared to provide respite from a fearful future, or be a source of hope for some.^30^, ^35^

#### 3.2.3 Loss of valued roles and activities, and the threat of a changing identity

The impact and fear of future impact of MS on valued roles and activities were frequently reported, reflecting the way that MS posed challenges to self‐perceptions and perceived identity, and difficulties in adapting to a changing body and altered capabilities.^38^ Guilt and shame associated with changing roles and abilities affecting family dynamics were expressed,^12^, ^30^, ^48^, ^63^ including an inability to provide for family through loss of employment and income.^33^, ^61^, ^64^, ^65^, ^66^, ^67^, ^68^, ^69^ Many studies highlighted the impact of MS on people's careers and employment.^7^, ^41^, ^48^, ^51^, ^57^, ^61^, ^70^, ^71^, ^72^, ^73^ The presence of supportive structures and environments at work were factors influencing whether people living with MS chose to remain in employment or not.^57^, ^73^

Disclosing or concealing a diagnosis was an important consideration in maintaining a sense of identity and avoiding stigma. Studies described concealing a MS diagnosis in the workplace^7^, ^30^, ^33^, ^57^, ^64^, ^73^ and social life.^7^, ^30^, ^33^, ^64^ Reasons included maintaining professional perceptions,^64^, ^73^ not wanting to be seen as different or disabled,^33^, ^40^, ^45^, ^57^, ^63^, ^64^, ^73^ and uncertainty about how others would respond.^7^, ^45^, ^57^, ^71^ When people living with MS did disclose their diagnosis, they reported both positive reactions, such as being supported and accommodated,^64^, ^73^, ^74^ and negative reactions, including having their work competency questioned^41^, ^57^, ^74^ and being treated dismissively.^57^, ^73^, ^74^

#### 3.2.4 Managing fatigue, and its impacts on life and relationships

Managing fatigue required constant planning and pacing of tasks to accommodate the anticipated fatigue‐related after effects.^10^, ^11^, ^29^, ^41^, ^47^, ^51^, ^58^, ^59^, ^64^ It was described in terms of its impact on work,^18^, ^49^, ^51^, ^70^, ^75^, ^76^ social life,^18^, ^45^, ^49^, ^51^, ^75^, ^76^, ^77^ family life^12^, ^18^, ^45^, ^49^, ^51^, ^52^, ^75^, ^76^, ^78^ and physical and psychological health.^14^, ^16^, ^47^, ^76^ People with MS described feelings of frustration about the limitations that fatigue imposed on their lives and the resulting loss of spontaneity.^18^, ^29^, ^47^ Knowing how to manage this was a source of confusion, with some people highlighting exercise^14^, ^16^, ^47^, ^76^ and diet^49^ as effective, and others attributing fatigue and relapse to incorrect, or too much, exercise.^14^, ^47^ Information and support to manage fatigue were found to be lacking for some, despite the significant impact it had on their lives.^18^, ^20^

People described emotional fatigue in relation to seeking information and support, and with interactions with health services.^38^, ^46^ This was influenced by an overall perceived lack of legitimacy^38^, ^40^ of invisible symptoms, and experiences of having to repeatedly explain or justify limitations to friends, family and workplaces,^31^, ^40^, ^46^, ^50^ to fight for needs from health professionals and government organizations,^31^, ^34^, ^38^, ^50^ and the need to regularly re‐establish relationships with rotating or changing health‐care providers.^38^, ^46^ Lack of community understanding about MS fatigue was recognized^22^ with people reporting feelings of guilt,^18^, ^51^ unreliability^18^, ^48^, ^51^ and being perceived as lazy^18^, ^20^, ^55^ when they were unable to meet work and social commitments due to fatigue.

#### 3.2.5 Adapting to life with MS

Strategies that people with MS used to adapt to MS ranged from, and oscillated between, denying the existence of their condition^45^ to total acceptance.^14^ Although denial of the diagnosis was experienced by some,^7^, ^30^ defiance in the sense of not letting MS and its impacts define identity, personal outlook and everyday life was most often expressed.^12^, ^30^, ^33^, ^49^, ^76^

For some people with MS, initial fears surrounding dependence on aids, such as wheelchairs, were replaced with acceptance and relief, as they facilitated adaptation to certain tasks, and assisted in maintaining independence.^29^, ^30^, ^71^, ^79^ Technology and devices were valued for enabling people to stay connected to society and community,^82^ follow online exercise programmes,^43^ monitor activity and fitness levels,^18^ and assist with daily living activities^53^ and work‐related tasks.^59^

The most frequently reported strategy for everyday coping was to draw on personal resources. Resources could include work,^64^, ^69^ spiritual faith,^33^, ^70^, ^81^ family support^10^, ^31^, ^82^ (including financial support to access care ^13^, ^66^) and social interaction, including engaging with other people with MS.^8^, ^16^, ^40^, ^47^, ^48^, ^82^

Our team members with MS affirmed the themes, and articulated some linkages across the themes, in narratives synthesized into I‐poems (Box 1). These poems contributed to the title of our paper.

##### BOX 1 Comments on experiential themes by people with MS

**Table:** 

Changing identity	Quest for knowledge	Uncertainty, quest for knowledge	Fatigue
I struggled with the identity issue for years. It struck at the heart of who I thought I was	There is not a lack of information out there – it is the opposite But it is not personalized and varies in quality and currency The onus is on you to take control and self‐educate	I still feel I have both too much and too little information	In 2011 I had transverse myelitis I spent most nights in intense, painful spasms I felt my level of fatigue increase and I am still fatigued

## 4 DISCUSSION

The five themes described in our review provide insight into people's experiences of MS. Most articles contained content which covered three or more domains and contexts, highlighting the interconnectedness of these experiences. For instance, experiences related to work, social and family life were rarely mentioned in isolation, and were closely linked to experiences of physical and psychological symptoms. Likewise, studies which included experiences related to receiving the diagnosis frequently referenced experiences with health services and health professionals – a pivotal point of contact, and one that is often vividly recalled, even many years after diagnosis.^85^

The two themes of *uncertain trajectories* and *quest for knowledge, expertise and understanding* are interwoven – with uncertainty itself related to an enacted quest for knowledge. People living with MS often experience long‐standing and unsettling symptoms before a diagnosis of MS is confirmed. Even when there is information available, information and support seeking may be complicated by a range of factors, including lack of integrated care, limited time with health‐care professionals, lack of referral to support services, or the knowledge, communication style and focus (eg pharmacological) of health‐care providers. This was particularly highlighted prior to and around the time of receiving a MS diagnosis.

The experiences described in this review suggest that the onus is on people with MS to take control and self‐educate, despite a lack of certainty about the very information that would enable them to do so (eg unknown aetiology and unpredictable prognosis, symptom trajectories and responses to treatment). This is challenging in the context of a diverse and information‐laden health landscape.^86^ In a qualitative study examining people with MS’ experiences, needs and preferences for integrating treatment information into decision making, participants described a desire for unbiased and up‐to‐date information. On the other hand, they reported an excess amount of information available, of which only a small amount was of relevance to them.^87^ Overall, participants expressed a desire to develop a ‘research partnership’ with health professionals to facilitate tailoring of information to meet their unique health needs.^87^ Acknowledging the presence of uncertainty with health professionals is the first step to achieve this aim.

Gheihman and colleagues^88^ propose that distinguishing between the many types and meanings of knowledge uncertainty is important in determining clinical management strategies. Clarifying *knowable* and *unknowable* forms and prioritizing techniques to address these are essential, in particular minimizing unnecessary uncertainties (knowable unknowns) through the provision of information. In line with the key experiences reported by people with MS and highlighted in this paper, this approach could help to address knowledge and intervention gaps for people with MS.

Fatigue is one of the most common and debilitating symptoms of MS, and managing this was described as a constant challenge by most participants in our review. Fatigue affects more than 80% of people with MS^89^ and is cited as the main reason why people with MS seek early retirement.^90^ Improving people with MS’ capacity to manage fatigue should be a priority for clinicians. While clinical trials have demonstrated some benefit associated with medication, physical activity and cognitive‐behaviour therapy,^89^ the experiences described in our review indicate that there is no one‐size‐fits‐all solution for fatigue.

A narrative review of apps developed to assist with MS self‐management found that most focused on physical and cognitive ability, and medication adherence, and few had been evaluated.^91^ However, repeated users of one interactive web‐based program, MSmonitor, reported improved ability to self‐manage fatigue and increased health‐related quality of life.^92^ Until recently, the needs of people with MS have not been accounted for in the development of apps.^93^ Patient and public involvement in research refers to the conduct of research ‘by’ or ‘with’ members of the public, rather than ‘for’ or ‘about’ them.^94^ Taking such an approach, a recent New Zealand study found that mobile technology provides an accessible and acceptable platform for the provision of interventions aimed at decreasing the impact and severity of fatigue in people with MS.^95^ Preliminary results of a web‐based survey of people with MS, using fatigue as a moderating influence, indicated that expectations of how helpful an app would be for self‐management, and social support was one indicator of acceptance.^96^ Other recent digital developments aimed at assisting people with MS to manage fatigue are involving people with MS.^91^, ^93^, ^95^

Patient experiences are infrequent outcomes in clinical trials of novel therapeutics. In their analysis of 16 pivotal MS drug trials relating to 8 of the recently introduced therapies, Gerardi et al^98^ found that all these drugs have to date been tested in 1‐ to 2‐year trials. Most drugs were compared to placebos but there have been no comparisons between established and recently introduced drugs. Two‐thirds of studies primarily examined relapse rate, with co‐primary examination of disability in two, but overall there was lack of consideration of patients’ preferences. Similarly, in their analysis of 29 Phase 3 trials of new disease‐modifying treatments for MS, Gehr and colleagues^99^ found that patients’ perspectives, including experiences of fatigue, cognitive impairment, pain, sleep disorders, loss of vision and spasticity, were mostly overlooked. They recommended designing studies that align with patients’ needs to ensure that results facilitate patient‐relevant outcomes. Our review supports this contention. Inclusion of patient preferences in outcomes of clinical trials would advance resolution of patient uncertainty, assist people with MS in making decisions and advance their quest for knowledge related to unknown impacts of treatments.

### 4.1 Limitations

A strength of our study is the incorporation of quality appraisal, which is not a requirement of scoping review approaches.^21^, ^98^ The exclusion of quantitative literature meant we were not able to include examination of patient‐reported experiences and outcomes elucidated through questionnaires. Insight into these, including quality of life, cost effectiveness, patient satisfaction and enablement, is essential to gain understanding of people's perceptions of both the process and outcome of health care.^101^ This paper does not address the grey literature about patient experiences, or works produced by patients themselves outside the scholarly literature, such as autobiographies and illness narratives. A few of the studies explicitly addressed people from low socio‐economic backgrounds. Some of the experiences described in this review may reflect the more individualistic cultures of the Global North, rather than more collectivist cultures. People with MS from North America and the United Kingdom accounted for 48% of the studies in this review, while there was only one study from Latin America^72^ and three from Asia.^20^, ^61^, ^67^

## 5 CONCLUSION

The majority of people in the studies included in this review expressed a determination to adapt to MS. The literature is replete with stories of survival and persistence, and a strong desire to remain engaged in society. The invisible aspects of MS, including fatigue, are often under‐appreciated by peers and clinicians. Our findings highlight the importance of the clinical partnerships between people with MS and their clinicians. In order to broaden their access to the ‘knowable form’ of knowledge underlying uncertainty, it is of critical importance to examine the long‐term risks and benefits of treatments, including patient‐reported outcomes, to enhance the capacities of people with MS and clinicians to make informed, person‐focused decisions.

## CONFLICT OF INTEREST

The authors declare that they have no competing interests.

## AUTHORS' CONTRIBUTIONS

CB, JD, AP, CL, JDr, KC, ME, VF were responsible for writing the original draft. JD, CP, CB, AP, KC,ME, CL, MC, JDr, VF, AB, HS, AT, AH were responsible for preparation, creation and presentation of the published work, specifically critical review, commentary or revision – including pre‐ or post‐publication stages. JD, CB and AP contributed to preparation, and creation and/or presentation of the published work, specifically visualization/ data presentation. JD and CP contributed to oversight and leadership responsibility for the research activity planning and execution, including mentorship external to the core team. JD contributed to management and coordination responsibility for the research activity planning and execution, and acquisition of the financial support for the project leading to this publication.

## Supporting information

Table S1

## Data Availability

Data available on request from the authors: the data that support the findings of this study are available from the corresponding author upon reasonable request. All data generated or analysed during this study are included in this published article (and its Supporting Information files).
