# Supplementary material for: ‘It struck at the heart of who I thought I was’: A meta‐synthesis of the qualitative literature examining the experiences of people with multiple sclerosis
Source: Health Expect. 2020 Jun 24;23(5):1007–27. doi: 10.1111/hex.13093 (PMC7696124; doi:10.1111/hex.13093)
Supplement: Supplementary file 1 — Table S1 [file HEX-23-1007-s001.docx]

Additional File 1: CASP Assessment

| **CASP QUESTIONS**  **Question 1:** Are the results valid?  **Question 2:** Is a qualitative methodology appropriate?  **Question 3:** Was the research design appropriate to address the aims of the research?  **Question 4:** Was the recruitment strategy appropriate to the aims of the research?  **Question 5:** Was the data collected in a way that addressed the research issue?  **Question 6:** Has the relationship between researcher and participants been adequately considered?  **Question 7:** Have ethical issues been taken into consideration?  **Question 8:** Was the data analysis sufficiently rigorous?  **Question 9:** Is there a clear statement of findings?  **Question 10:** How valuable is the research?  **Y =** YES **N =** NO **CT =** CAN’T TELL | | | | | | | | | | | |
| --- | --- | --- | --- | --- | --- | --- | --- | --- | --- | --- | --- |
|  |  |  |  |  |  |  |  |  |  |  |  |
| **Author** | **Year** | **Q1** | **Q2** | **Q3** | **Q4** | **Q5** | **Q6** | **Q7** | **Q8** | **Q9** | **Q10** |
| Turpin et al | 2018 | Y | Y | Y | Y | Y | **CT** | Y | Y | Y | Y |
| Tabuteau-Harrison et al | 2016 | Y | Y | Y | **N** | Y | **CT** | Y | Y | Y | Y |
| Strickland et al | 2017 | Y | Y | Y | Y | Y | **CT** | Y | Y | Y | Y |
| Stone et al | 2013 | Y | Y | Y | Y | Y | Y | Y | Y | Y | Y |
| Stern & Goverover | 2018 | Y | Y | Y | Y | CT | Y | Y | Y | Y | Y |
| Stennet et al | 2018 | Y | Y | Y | **CT** | Y | Y | Y | Y | Y | Y |
| Soundy et al | 2012 | Y | Y | Y | Y | Y | **N** | CT | Y | Y | Y |
| Sosnowy | 2014 | Y | Y | Y | Y | Y | **N** | Y | **N** | **CT** | Y |
| Smith et al | 2015 | Y | Y | Y | Y | Y | **N** | Y | Y | Y | Y |
| Smith et al | 2011 | Y | Y | Y | Y | Y | Y | Y | Y | Y | Y |
| Skovgaard et al | 2014 | Y | Y | Y | Y | Y | **CT** | **CT** | Y | Y | Y |
| Skovgaard et al | 2014 | Y | Y | Y | Y | Y | N | **CT** | **CT** | Y | Y |
| Skar et al | 2014 | Y | Y | Y | Y | Y | N | Y | Y | Y | Y |
| Russell et al | 2018 | Y | Y | Y | Y | Y | **CT** | Y | Y | Y | Y |
| Rintel et al | 2012 | Y | Y | Y | Y | Y | Y | **CT** | Y | Y | Y |
| Riazi et al | 2012 | Y | Y | Y | **CT** | Y | **CT** | Y | Y | Y | Y |
| Pretorius & Joubert | 2014 | Y | Y | Y | Y | Y | **CT** | Y | Y | Y | Y |
| Plow & Finnlayson | 2012 | Y | Y | Y | **CT** | Y | **CT** | **CT** | Y | Y | Y |
| Ploughman et al | 2012 | Y | Y | Y | Y | Y | Y | Y | Y | Y | Y |
| Yilmaz et al | 2017 | Y | Y | Y | Y | Y | **CT** | Y | **CT** | Y | Y |
| Willson et al | 2018 | Y | Y | Y | Y | Y | Y | Y | Y | Y | Y |
| Vijayasingham et al | 2017 | Y | Y | Y | Y | Y | **CT** | Y | Y | Y | Y |
| van der Meide | 2018 | Y | Y | Y | Y | Y | Y | Y | Y | Y | Y |
| Payne & Kathryn | 2010 | Y | Y | Y | **N** | Y | **CT** | Y | Y | Y | Y |
| Parton et al | 2018 | Y | Y | Y | Y | Y | **CT** | Y | Y | Y | Y |
| Parton et al | 2017 | Y | Y | Y | Y | Y | **N** | Y | Y | Y | Y |
| Olsson et al | 2010 | Y | Y | Y | Y | Y | Y | Y | Y | Y | Y |
| Olsson et al | 2011 | Y | Y | Y | Y | Y | **N** | Y | Y | Y | Y |
| Mozo-Dutton et al | 2012 | Y | Y | Y | Y | Y | **CT** | Y | Y | Y | Y |
| Morley et al | 2013 | Y | Y | Y | Y | Y | **CT** | Y | Y | Y | Y |
| Moriya & Suzuki | 2011 | Y | Y | Y | Y | Y | **CT** | Y | Y | Y | Y |
| Moriya & Kutsumi | 2010 | Y | Y | Y | **CT** | Y | **CT** | Y | Y | Y | Y |
| Meade et al | 2016 | Y | Y | **CT** | Y | Y | **CT** | Y | **CT** | Y | Y |
| Sharifi & Abbaszadeh | 2016 | Y | Y | Y | Y | Y | Y | Y | Y | Y | Y |
| Senders et al | 2016 | Y | Y | Y | Y | Y | **CT** | Y | Y | Y | Y |
| Masoudi et al | 2015 | Y | Y | Y | Y | Y | **N** | Y | Y | Y | Y |
| Maghsoodi & Mohammadi | 2018 | Y | Y | Y | Y | Y | **N** | Y | Y | Y | Y |
| Lynd et al | 2018 | Y | Y | Y | Y | Y | **N** | Y | Y | Y | Y |
| Lynass and Gillon | 2017 | Y | Y | Y | Y | Y | Y | Y | Y | Y | Y |
| Lohne et al | 2010 | Y | Y | Y | Y | Y | **N** | Y | Y | Y | Y |
| Lexell et al | 2011 | Y | Y | Y | Y | Y | **N** | Y | Y | Y | Y |
| Lee Mortensen & Rasmussen | 2017 | Y | Y | Y | Y | Y | **N** | Y | Y | Y | Y |
| Hunt et al | 2014 | Y | Y | Y | Y | Y | Y | Y | Y | Y | Y |
| Hosseini et al | 2016 | Y | Y | Y | Y | Y | **N** | Y | Y | Y | Y |
| Newland et al | 2012 | Y | Y | **CT** | Y | Y | **CT** | Y | Y | Y | Y |
| Harrison et al | 2015 | Y | Y | Y | Y | Y | Y | Y | Y | Y | Y |
| Encarnação et al | 2016 | Y | Y | Y | Y | Y | **CT** | Y | Y | Y | Y |
| Dlugonski et al | 2012 | Y | Y | Y | Y | Y | Y | **CT** | Y | Y | Y |
| Dennison et al | 2011 | Y | Y | Y | Y | Y | **CT** | Y | Y | Y | Y |
| Dennison et al | 2016 | Y | Y | Y | Y | Y | **CT** | Y | Y | Y | Y |
| Deghan-Nayeri et al | 2018 | Y | Y | Y | Y | Y | **N** | Y | Y | Y | Y |
| Deghan-Nayeri et al | 2017 | Y | y | y | y | y | **N** | Y | Y | Y | Y |
| Deghan-Nayeri et al | 2018 | Y | Y | Y | Y | Y | **N** | Y | Y | Y | Y |
| de Ceuninck et al | 2016 | Y | Y | Y | Y | Y | **N** | Y | Y | Y | Y |
| de Ceuninck et al | 2017 | Y | Y | Y | Y | Y | **N** | Y | Y | Y | Y |
| Knaster et al | 2011 | Y | Y | Y | Y | Y | **N** | Y | Y | Y | Y |
| Kirk-Brown & Van Dijk | 2014 | Y | Y | Y | Y | Y | **N** | Y | Y | Y | Y |
| Kayes et al | 2011 | Y | Y | Y | Y | Y | **N** | Y | Y | Y | Y |
| Cowan et al | 2018 | Y | Y | Y | Y | Y | Y | Y | Y | Y | Y |
| Coenen et al | 2011 | Y | Y | Y | Y | Y | Y | Y | Y | Y | Y |
| Chard | 2017 | Y | Y | Y | Y | Y | **N** | Y | Y | Y | Y |
| Brunn Helland et al | 2015 | Y | Y | Y | Y | Y | Y | Y | Y | Y | Y |
| Browne et al | 2015 | Y | Y | Y | Y | Y | Y | Y | Y | Y | Y |
| Bogosian et al | 2017 | Y | Y | Y | Y | Y | **N** | Y | Y | Y | Y |
| Bogenscutz et al | 2016 | Y | Y | Y | Y | Y | **N** | Y | Y | Y | Y |
| Blundell Jones et al | 2014 | Y | Y | Y | Y | Y | Y | Y | Y | Y | Y |
| Asanao et al | 2015 | Y | Y | Y | Y | Y | **N** | Y | Y | Y | Y |
| Giovannetti et al | 2017 | Y | Y | Y | Y | Y | **N** | Y | Y | Y | Y |
| Ghafari et al | 2014 | Y | Y | Y | **CT** | Y | Y | Y | **CT** | Y | Y |
| Ghafari et al | 2015 | Y | Y | **CT** | **CT** | Y | Y | Y | Y | Y | Y |
| Gaskill et all | 2011 | Y | Y | Y | **CT** | Y | Y | **CT** | Y | Y | Y |
| Frost et al | 2017 | Y | Y | Y | Y | Y | Y | **CT** | Y | Y | Y |
| Fallahi et al | 2014 | Y | Y | Y | Y | Y | **N** | Y | Y | Y | Y |
| Anderson et al | 2013 | Y | Y | Y | Y | Y | **CT** | **N** | CT | Y | Y |
| Aminian et al | 2017 | Y | Y | Y | Y | Y | **CT** | Y | Y | Y | Y |
| Al-Sharman et al | 2018 | Y | Y | Y | Y | Y | Y | Y | Y | Y | Y |
| Adamson et al | 2018 | Y | Y | Y | Y | Y | **CT** | **CT** | Y | **CT** | Y |
